# Sex Matters: Hormonal and Chromosomal Determinants of Autoimmunity and Anti‐Cancer Immunity Across the Lifespan

**DOI:** 10.1111/imr.70096

**Published:** 2026-01-24

**Authors:** Christian G. Bustillos, Esther M. Peluso, Sophia L. Cha, Melissa G. Lechner, Maureen A. Su

**Affiliations:** ^1^ Department of Microbiology, Immunity, and Molecular Genetics UCLA David Geffen School of Medicine Los Angeles California USA; ^2^ Department of Molecular and Medical Pharmacology UCLA David Geffen School of Medicine Los Angeles California USA; ^3^ UCLA/California Institute of Technology Medical Scientist Training Program UCLA David Geffen School of Medicine Los Angeles California USA; ^4^ Department of Medicine UCLA David Geffen School of Medicine Los Angeles California USA; ^5^ Department of Pediatrics UCLA David Geffen School of Medicine Los Angeles California USA

**Keywords:** autoimmunity, cancer, hormones, sex, X chromosome, Y chromosome

## Abstract

Sex plays a key role in shaping both anti‐cancer immunity and autoimmunity. Biological factors underlying sexual dimorphism have now been identified in multiple aspects of anti‐cancer immunity and autoimmunity. These factors include sex differences in hormone levels, chromosome complement, and expression of the long non‐coding RNA XIST. In this review, we discuss recent advances delineating how these differences alter immune responses against cancer and autoimmune responses against healthy tissues. Moreover, we now understand that hormone levels change (e.g., in mini‐puberty, menopause, and andropause) and that somatic alterations in chromosomal complement accumulate (e.g., loss of Y [LOY] chromosome) across the lifespan. We also include here a discussion of how these changes affect anti‐cancer immunity and autoimmunity across a lifetime. These recent advances will set the stage for identifying immunotherapeutic approaches that optimize anti‐cancer immunity while controlling the autoimmune responses.

## 1 Introduction

The immune response to cancer and autoimmunity are closely linked. This is illustrated by the high rates of autoimmune side effects that occur with treatments that boost anti‐cancer immunity [1]. Approximately 40% of cancer patients treated with immune checkpoint immunotherapies developed immune related adverse events (IRAEs). Moreover, better cancer outcomes were reported in patients who developed IRAEs and better outcomes with reduced incidence of melanoma in individuals who developed vitiligo [2, 3, 4]. In both anti‐cancer immunity and autoimmunity, sex plays a critical role as a biologic variable in determining incidence and outcomes. Here we will review recent progress in understanding hormonal and chromosomal differences that underlie sex differences in autoimmunity and anti‐cancer immunity. Because hormone levels change and chromosomal changes accumulate throughout the lifespan, the impact of age on sex differences is also discussed in this review. Understanding these differences will pave the path to developing new approaches for modulating the immune response to benefit patients with cancer, autoimmunity, and both cancer and autoimmunity.

## 2 Sex Hormones Across the Lifespan and Their Effects on Autoimmunity

Autoimmune diseases demonstrate a striking sex bias with women disproportionately affected compared to men. Approximately 80% of patients treated for autoimmunity are women, with diseases such as Sjogren's syndrome, Hashimoto's thyroiditis, systemic lupus erythematosus (SLE), scleroderma, myasthenia gravis, and rheumatoid arthritis (RA) demonstrating the greatest female to male bias [5, 6]. In addition to chromosomal and environmental differences between men and women, sex hormones are powerful modulators of immune responses. Sex hormones derive primarily from the gonads, namely ovaries in females and testes in men, and to a lesser extent the adrenal glands, liver, and adipose tissue. The three main sex hormone classes in humans are estrogens, androgens, and progestogens, which classically act on their respective nuclear steroid receptors to regulate target gene expression.

While estrogens are generally thought to be proinflammatory in autoimmunity, there is also strong evidence that the opposite might be true. For example, in patients with multiple sclerosis (MS), estrogens improve symptoms and in RA low estrogen is associated with increased RA flares [7, 8, 9]. The estrogen class of molecules consists of estrone, estradiol, and estriol which act on nuclear and membrane‐bound estrogen receptors (ER). Nuclear receptors are ERɑ (*Esr1*) and ERβ (*Esr2*), and membrane‐bound ER is the G‐protein‐coupled estrogen receptor GPER1 (*GPER1*). ER signaling leads to genomic changes and signaling pathway modulation that lead to specific cell and tissue alterations. ER can localize with other transcription factors including AP‐1, SP‐1, and NF‐κB to modulate immune gene expression. In contrast, GPER1 acts via second messengers and signaling cascades [10].

The lower prevalence of autoimmune disease in men is attributed in part to the protective effects of androgens. In a longitudinal study that followed 500,000 men in the U.S., untreated hypogonadism was associated with the development of RA and SLE [11]. Androgens (primarily testosterone or dihydrotestosterone) freely cross the cellular membrane and bind to AR in the cytoplasm where the complex then translocates into the nucleus, dimerizes, and binds to androgen response elements (ARE) on the promoter or enhancer regions of target genes. DNA‐independent actions of AR through second messenger cascades such as ERK, Akt, and MAPK pathways have also been reported [12].

Sex hormone levels naturally fluctuate with age and health status, which may in turn result in changes in immune responses linked to sex hormones. For instance, estrogens have critical roles in immune function and can drastically vary in levels throughout adulthood, such as during ovulation (the female menstrual cycle), periods of physiologic stress (hypothalamic hypogonadism), and elevated levels during pregnancy. Indeed, the end of the reproductive phase in adulthood is punctuated by the abrupt fall in estrogen production with menopause. This section will discuss the impact of sex hormones in the adaptive immune responses across the lifespan to highlight windows of protection and vulnerability for autoimmunity (Table 1). In the following sections we will review recent studies investigating the effect of estrogens, androgens, and progesterone on adaptive immune cell development throughout the lifespan.

**TABLE 1:** Sex hormones regulate peripheral immune function and risk for autoimmunity across the lifespan.

	Androgens	Estrogens
Mini‐puberty	Brief 3–6‐month surge of pituitary and sex hormones [13] Higher CD4^+^ T cells in female blood [14] Higher CD8^+^ T cells in male blood [14]	
Childhood	Tonic inhibition of HPG axis by GnRH Estrogen and androgen levels are relatively equal	
Puberty and adulthood	Initiated by the pulsatile release of GnRH that stimulates pituitary LH/FSH release to stimulate gonadal androgen and estrogen production	
	Androgen levels in men are constant and dampen immune responses Directly upregulate Ptpn22 to decrease TCR strength and protect against mouse models of T1D and SLE [15] Directly modulate H3K27Me3 methylation marks near glutamine transport proteins in Th17 cells to alter metabolic pathways [16] AR enhances Treg suppressive function and increases Treg/Th2 ratio in allergic inflammation [17] AR can promote histone H4 acetylation near the *Foxp3* locus [18] Androgens regulate bone marrow development of B cells through decrease *BAFF* [19, 20]	Levels of estrogen vary during the menstrual cycle, physiologic stress, and disease states Females have greater methylation of EREs near TCR, estrogen receptor, and androgen receptor signaling pathways [21] Puberty is associated with a shift from innate to adaptive immune gene expression [22] Estradiol directly promotes Th1 responses [23] Estrogen's effect on T cells are context dependent: ERα and ERβ have divergent functions depending on disease model and tissue context ERα promotes inflammation in colitis: loss in CD4^+^ T cells decreases IFNy and IL‐17A while increasing Tregs [24] ERα supports T cell activation: loss in CD4^+^ T cells impairs proliferation and activation [24] ERβ has a protective role in colitis: global Erβ deletion decreases Treg levels and worsens colitis [25] ERα deficiency increases Tfh responses [26] ERβ promotes pathogenic Th17 response in autoimmune thyroiditis [27] Estrogen is protective in EAE through ERα and ERβ [28, 29, 30] Estrogens contribute to B cell pathogenesis via B cell activation, antibody production and apoptosis pathway modulation [31, 32, 33]
Menopause and andropause	Can be medically induced and causes sudden loss of estrogen or androgen signaling (e.g., cancer or endometriosis treatment) Equalization in autoimmunity risk in males and females Aging is associated with immuno‐senescence	
	Steady decline of androgen production Associated with increased risk of autoimmunity in men [34]	Sudden loss of estrogen and progesterone production Loss of estrogens associated with immuno‐senescence and greater level of proinflammatory cytokines [35]

*Note:* Blue boxes are specific to androgens. Pink boxes are specfic to estrogens. Grey boxes are not specific to androgens or estrogens.

### 2.1 In Utero

Sex hormone exposure begins in utero. While relatively protected from external stimuli during in utero growth, the fetus receives maternal sex hormones and immunologic signals via the placenta that may influence the developing fetal immune system with long‐lasting effects. The fetus is exposed to maternal placental sex hormones including estradiol, estriol, and progesterone, which progressively increase during pregnancy to peak during the third trimester. The placenta provides passive immunity to the developing fetus through antibodies and cytokines that can influence fetal immune development. In mouse models, maternal asthma during pregnancy increases glucocorticoid signaling that primes and epigenetically imprints fetal Group 2 Innate Lymphoid Cells (ILCs) in the lung for long lasting hyperresponsiveness to allergens and subsequent asthma in adult life [36]. This provides evidence that steroid hormones can have effects on fetal immune system development, although sex steroids in particular have not directly been studied. Because innate and adaptive lymphoid cells begin development as early as 8–12 weeks postconception, it is plausible that estrogen and androgen levels can imprint immune cells during fetal development to increase risk toward autoimmunity later in life, though direct evidence is limited [37, 38].

In addition to maternal hormones, the fetal gonads begin to develop and differentiate at 6 weeks gestational age. In males, testosterone hormone production begins around 9–10 weeks gestational age, and in females, the adrenal gland is the predominant producer of DHEA and estrogen precursors, which are converted to estrogen by the placenta [39, 40, 41]. Fetal anomalies in development can result in abnormal gonads and fetal sex hormone excess or deficiency. For example, genetic pathogenic variants leading to the classic form of congenital adrenal hyperplasia (CAH) lead to an excess of androgens that begins in utero. How these differences in sex hormone exposure in utero may alter development of the immune system is not fully understood.

### 2.2 Childhood

Sex hormones in childhood typically remain at low levels, with the exception of a brief period early in life (Table 1). In the early post‐natal period, infants experience a brief surge in pituitary and sex hormones termed ‘mini‐puberty’. This 3–6 month period in both male and female infants reflects re‐activation of the hypothalamic–pituitary‐gonadal (HPG) axis in rebound from the high levels of maternal placental sex hormones after birth that suppress the fetal axis through negative feedback [13]. In this stage hypothalamic gonadotropin releasing hormone (GnRH) secretion increases and stimulates the pituitary to release luteinizing hormone (LH) and follicle stimulating hormone (FSH) to increase gonadal production of estrogens and androgens. During this period, few sex differences in the immune system have been analyzed, but peripheral blood from female infants displayed higher levels of CD4^+^ T cells, whereas male infants had higher CD8^+^ T cell levels [14]. Thus, it has been suggested that infant mini‐puberty represents a window of opportunity where the immune system undergoes dramatic changes that might lead to later susceptibility to autoimmunity.

During later childhood, the levels of estrogen and androgens remain relatively equal due to the tonic inhibition of the HPG axis by GnRH. In this life stage, sex differences in autoimmunity are less pronounced. Notably, a few diseases that show a female predisposition in childhood include oligoarticular Juvenile Idiopathic Arthritis (JIA), autoimmune thyroid disease, and Juvenile dermatomyositis [42, 43]. Evidence exists that in childhood onset SLE, there is a suppression of androgen steroidogenesis and reduced ovarian volumes [44]. Thus, alterations in hormone levels might predispose to early onset autoimmunity. While many of these autoimmune diseases have strong hormonal and chromosomal mechanistic studies demonstrating their sex bias, it is possible that these diseases manifest during prepuberty, but they are a result of sex hormone signaling in fetal and mini‐puberty life.

### 2.3 Puberty

As with mini‐puberty discussed above, puberty is initiated by the pulsatile release of GnRH from the hypothalamus that signals the pituitary gland to release gonadotropins FSH and LH. FSH and LH act on either the testes or ovaries to stimulate the production of androgens or estrogens, leading to the marked rise in these sex hormones associated with puberty (Table 1). This onset of puberty occurs around 9–14 years in males and 8–12 years in females [45]. This period coincides with the emergence of the female prevalence of autoimmunity seen in diseases such as SLE, MS, Hashimoto's thyroiditis, and Grave's disease [46, 47, 48]. During puberty, the rise in sex hormones affects key changes in central and peripheral immune processes that are thought to contribute to the female prevalence of autoimmunity.

#### 2.3.1 Central Immune Tolerance

In T cell development, early thymic progenitors migrate from the bone marrow to the thymus. Within the thymus these developing T cells (thymocytes) interact with thymic epithelia cells (TECs) to undergo positive selection to ensure a functioning T cell receptor and subsequent negative selection to remove potentially self‐reactive T cells from maturing and moving to peripheral tissues. Both TEC and developing thymocytes express sex hormone receptors [estrogen receptor alpha (ERα), estrogen receptor beta (ERβ), and G‐protein‐coupled estrogen receptor 1 (GPER1), androgen receptor (AR), and progesterone receptor (PR)] and have shown response to estrogens, androgens, and progesterone [49]. Through effects on positive and negative selection, these sex hormones shape central immune tolerance and subsequently the propensity for autoimmune disease.

The rise of sex hormones during puberty corresponds with a period of thymic involution. Estrogens promote thymic involution by action of ERɑ and GPER1 on the stromal compartment of the thymus and as a result blockade of estrogen synthesis results in increased thymic size [50, 51, 52]. Within the thymic cortex, estradiol treatment acts through ERɑ to inhibit NF‐κB signaling in CD25‐ double negative thymocytes to block further maturation, whereas GPER1 activity increases apoptosis in TCRβ‐low thymocytes [50]. Androgens also act to decrease thymic output through effects on positive selection. Global knockout of AR in mice increased the quantity of double negative, double positive, and single positive thymocytes [53]. This effect is most likely due to action on TECs because TEC‐specific AR knockout increased thymic weight and thymocyte development, whereas T cell‐specific AR deletion did not affect these outcomes [19, 53]. Other studies have shown that androgens can decrease thymocyte positive selection (lineage commitment) by limiting the cellularity of cortical TECs and by directly inhibiting Notch ligand delta like 4 (DLL4), which is critical for T cell commitment and differentiation [54, 55]. Consistent with these findings in animal models, hypogonadism in men increased thymic output [56]. In summary, both estrogens and androgens influence positive selection in the thymus and thymic T cell output.

**FIGURE 1:**
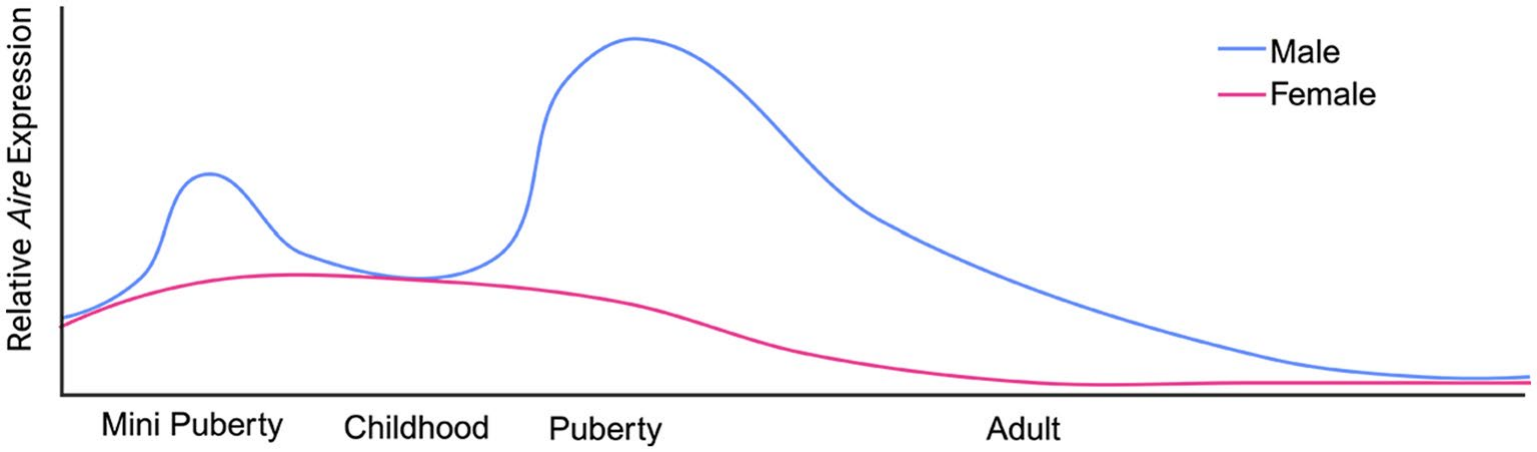
Relative *AIRE* expression in males versus females across the lifespan. Male neonates experience a brief androgen‐driven surge in *AIRE* expression during mini puberty. After this period, AIRE levels are similar between sexes until puberty. At puberty, androgens increase *AIRE* transcription while estrogens promote CpG methylation of the Aire promoter to decrease transcription. AIRE levels progressively decline in both sexes throughout adulthood.

Negative selection is another critical step in thymocyte development that impacts autoimmune disease risk. Autoimmune regulator (AIRE) is a key transcription factor that acts in medullary thymic epithelial cells (MTEC) to increase the expression of tissue‐specific self‐antigens and their presentation in MHC molecules to developing thymocytes. This presentation of self‐antigens by MTECs to developing thymocytes is critical to T cell negative selection and prevents the release of autoreactive T cells into the periphery. Multiple researchers have investigated sex differences in thymic changes in *Aire* expression over different life stages (Figure 1). First, Zhu et al. measured increased levels of *Aire* in MTECs from male newborns compared to female newborns during the first week of life and at five months, representing the window of mini‐puberty [57]. Interestingly, this sex difference in thymic *Aire* expression is lost during later childhood, when sex hormone levels generally equalize between males and females, but reemerges during puberty [58]. During puberty, the presence of androgens increased *Aire* gene expression in MTECs to increase negative selection and decreased the pool of self‐reactive T cells and development of autoimmunity in a mouse model of EAE [57]. By contrast, estrogens act via ERɑ to increase CpG methylation sites of the *AIRE* promoter region and thereby decrease AIRE‐mediated tissue specific antigen expression [58]. This resulted in decreased negative T cell selection in the thymus and increased escape of self‐reactive T cells. Indeed, treatment of male mice with estradiol increased experimental autoimmune thyroiditis (EAT) [58]. Estrogens were shown to further attenuate thymocyte negative selection by decreasing the expression of MHC molecule HLA‐DR in TECs [59].

#### 2.3.2 Peripheral Tolerance

Hormonal alterations during puberty can also lead to gene expression and epigenetic changes in peripheral blood immune cells. In a recent study, PBMCs from males and females pre‐ and post‐puberty were analyzed for epigenetic changes and researchers identified differentially methylated regions in genes of males and females [21]. Females had the greatest increase in methylation pattern changes near known estrogen responsive elements (EREs) [21]. In this study, genes with the greatest changes in methylation pattern were enriched in T cell receptor (TCR), ERβ, and AR signaling [21]. Similarly, in a longitudinal cohort of pre‐ and post‐puberty males and females with asthma, Resztak et al. collected gene expression data that demonstrated a shift from innate to adaptive immunity in females via increased gene enrichment for TCR signaling pathways with the onset of puberty [22]. These changes in methylation patterns and gene signaling could underlie the emergence of female predominance in autoimmunity with the onset of hormonal changes during puberty.

### 2.4 Adulthood

During adulthood, females and males experience the peak levels of their respective sex hormones. In addition to the persistence of sex hormone effects on central immune tolerance discussed above, adulthood is marked by sex‐specific immune responses due to the action of sex hormones on peripheral adaptive and innate immune cell populations (Table 1). Here, we will focus on recent studies examining the effects of estrogens and androgens on adaptive immune cells and the consequence of these changes on the prevalence and severity of autoimmune disease.

#### 2.4.1 Estrogens

##### 2.4.1.1 Estrogen Effects on T Cell Mediated Autoimmunity

In females, T cells are exposed to estrogens in the periphery, which can play a major role in T cell function and activation. In the periphery, estrogens are considered to be either pro‐ or anti‐inflammatory depending on the receptor they act on and the concentration of the hormone. Within CD8^+^ T cells, ERα and ERβ have relatively equal mRNA and protein expression [60]. CD4^+^ T cells, on the other hand, have greater ERα expression compared to Erβ [60].

Over 30 years ago, Fox et al. demonstrated that estradiol can bind to ERs and subsequently localize to EREs in the promoter region of interferon gamma (*Ifng*) to increase gene expression in CD4^+^ T cells [23]. Thus, it has long been known that estrogen can promote increased T helper 1 (Th1) immune responses. More recently, the role of ERα has been studied in other effector T cell subtypes, but the results of these studies suggest that the role of estrogens is nuanced and likely depends on the context in which it is studied. In a mouse model of colitis, researchers found that ERα deletion in CD4^+^ T cells led to decreased colitis severity, accompanied by fewer IFNy^+^ and interleukin (IL)‐17A^+^ proinflammatory CD4^+^ T cells and increased CD4^+^ T regulatory cells [24]. The authors further demonstrated that loss of ERα in CD4^+^ T cells led to decreased proliferation and activation, suggesting a pro‐inflammatory role of ERα signaling in this context. On the other hand, global loss of ERβ in a mouse model of colitis led to decreased levels of Treg cells and increased severity of disease, suggesting an opposite effect in CD4^+^ T cells from ERα in Tregs [25]. Further, deletion of ERα in CD4^+^ T cells in mice spontaneously increased T follicular helper cell number percent in the spleen and mesenteric lymph node and autoantibody production in aged mice. To test the functional consequences of ERα deficiency in CD4^+^ T cells, the authors further immunized mice with sheep red blood cell and showed an increase in germinal center formation in female mice with ERα deficiency, suggesting a suppressive effect of estrogens in CD4^+^ T cells in a different autoimmune context [26]. On the other hand, it is also possible that these observations are due to estrogen effects in skewing CD4^+^ T cell differentiation toward different subsets rather than a global inhibitory effect.

In models of other female‐sex biased autoimmune diseases we find additional evidence of a pro‐inflammatory role of estrogens. Another strikingly sex‐biased autoimmune disease is autoimmune thyroiditis (AIT). In a mouse model of experimental autoimmune thyroiditis (EAT), investigators first reduced endogenous estrogens via ovariectomy and then selectively activated ERα or ERβ with specific agonists. Activation of ERβ worsened EAT. The authors further demonstrated that ERβ engages with NF‐κB directly via pull down assays to increase IL‐17 and IL‐21 gene expression in CD4^+^ T cells of mice with AIT [27]. On the other hand, in a mouse model of autoimmune experimental encephalitis (EAE), which recapitulates MS, estradiol inhibited CD4^+^ Th1 and Th17 cell differentiation via an ERα‐dependent mechanism and was protective for autoimmunity [28]. Another EAE study showed that estradiol conferred protection through ERβ dependent signaling in microglia, leading to reduced NF‐κB activity and decreased nitric oxide production by microglia and CD3^+^ T cells [29, 30]. Taken together, these data demonstrate the possible pro‐ and anti‐inflammatory role of estrogens on CD4^+^ T cells across different autoimmune disease models.

##### 2.4.1.2 Estrogen Effects on B Cell Mediated Autoimmunity

B cells contribute to disease pathogenesis in several female predominant autoimmune diseases, such as SLE, MS, and AIT. Early work showed that estrogen treatment in B cells modulates apoptotic and survival genes (*CD22, SHP1, BCL2*) to promote increased survival and activity of autoreactive B cells [31]. In vitro studies further demonstrated that B cells express higher levels of ERβ than ERα [32]. In mouse models of SLE, B cell specific deletion of ERα reduced B cell activation, pathogenic autoantibody production, and overall disease severity [33]. In patients with SLE, disease flares and increased disease severity are observed during pregnancy when estrogen and progesterone levels are high, suggesting a pro‐inflammatory role in SLE that warrants further study [61]. In conclusion, the role of estrogens in autoimmunity is nuanced and likely depends on the disease‐specific context.

#### 2.4.2 Androgens

##### 2.4.2.1 Androgen Effects on T Cell Mediated Autoimmunity

Prior work has shown that androgens can dampen T cell function by altering TCR signaling, cellular metabolism, and the balance of CD4^+^ T cell subsets. In mouse models of SLE and autoimmune diabetes (T1D), Lee et al. identified a mechanism in which androgens attenuate T cell activation through upregulation of Protein tyrosine phosphatase non‐receptor type 22 (Ptpn22), a negative regulator of TCR signaling [15]. They demonstrated that liganded AR bound a conserved ARE within the *Ptpn22* regulator region, increasing its transcription. Elevated Ptpn22 expression reduced TCR signaling strength, resulting in a shift toward memory precursor effector cells, and loss of Ptpn22 increased autoimmune severity in mouse models of T1D and SLE.

Androgens also influence CD4^+^ T cell metabolism in ways that influence differentiation toward autoimmune prone lineages. In allergic airway inflammation, Chowdhury et al. reported that male Th17 cells relied less on glutaminolysis than female Th17 due to AR‐dependent modulation of repressive H3K27Me3 methylation marks near glutamine transport proteins [16]. These findings align with prior studies done in mouse models of asthma, where androgens have been shown to suppress ILC2 cytokine production and dampen Th2 and Th17 (IL17A) immune responses [62, 63]. More recently, Gandhi et al. found that AR activity enhanced Treg suppressive function and increased the Treg/Th2 ratio in mouse models of allergic airway inflammation [17]. While the study did not identify a direct action of AR on Tregs, earlier work showed that AR can promote histone H4 acetylation near the *Foxp3* locus, supporting an epigenetic mechanism of AR activity on DNA [18]. Collectively, these recent studies demonstrate that androgens can modulate multiple T cell pathways including TCR signaling, metabolism, and lineage differentiation to attenuate immune responses in the context of autoimmunity.

##### 2.4.2.2 Androgen Effects on B Cell Mediated Autoimmunity

In B cells, the AR expression has primarily been described in developing B cells and bone marrow stromal cells that facilitate B cell maturation [20]. Thus, androgens regulate bone marrow B cell lymphopoiesis, and a recent study by Wilhelmson et al. showed that testosterone attenuated the B cell survival factor BAFF (B cell activating factor). Castration, with loss of the testes and gonadal androgens, increased serum BAFF levels and the quantity of splenic B cells [19]. In line with an immunosuppressive role on B cells, men with Klinefelter syndrome (XXY), who have lower levels of androgens compared to unaffected men (XY), have an increased risk for female predominant autoimmune diseases, with an 18–19‐fold risk of SLE and Sjogren's syndrome, and notably increased B cell numbers and immunoglobulins [64, 65, 66]. Furthermore, testosterone replacement therapy in such patients has also been shown to decrease B cell numbers [64]. Similarly, gonadotropin treatment in patients with idiopathic hypogonadotropic hypogonadism decreased T and B cell counts [66]. In summary, androgens demonstrate a suppressive role on B cell function and antibody production, though data is needed in the context of specific autoimmune diseases.

### 2.5 Pregnancy

Abrupt changes in hormone levels, such as during pregnancy and monthly cyclical changes can help elucidate their protective or aggravating effects with respect to autoimmunity. The maternal hormone changes seen during pregnancy, a period requiring increased maternal immune tolerance for the developing fetus, are associated with a less inflammatory state in many autoimmune diseases. For example, patients with MS, rheumatoid arthritis (RA), and Graves' thyroid disease see relief in the third trimester as estrogen and progesterone are in their highest concentration, with worsening of symptoms postpartum [67, 68, 69]. Progesterone, the hormone named to promote gestation, is responsible for many of the immune shifts observed during pregnancy and is generally considered anti‐inflammatory. Progestins, such as progesterone, act via binding of intracellular PR (PRA and PRB) but can also signal through membrane PRs and cytosolic pregnane X receptor (PXR) and glucocorticoid receptors. Progestins have a minor contribution to central immune tolerance and a greater importance in peripheral immunosuppression likely critical for maternal‐fetal tolerance.

#### 2.5.1 Progesterone

##### 2.5.1.1 Central Tolerance

While progesterone treatment induces thymic involution, it is likely not a major contributor to age related thymic involution. More likely, thymic involution with increasing progesterone promotes maternal fetal tolerance. Nuclear progesterone levels increase during pregnancy and mice with loss of intracellular PR are resistant to thymic involution during pregnancy [70, 71]. Similar to androgens and estrogens, the main target of progesterone in the thymus is TECs. Specifically, during pregnancy, there is a decrease in the number of all non‐lymphoid cell types in the thymus and loss of early thymic progenitor homing to the thymus through loss of homing chemokines such as CCL25 and CXCL12 expressed by epithelial cells [72]. In vitro experiments with human mTEC show that exogenous estrogen administration decreased *Aire* expression [58]. In contrast, Ahn et al. show that within the medulla of the thymus, AIRE and AIRE‐dependent genes (*Hbby*, *Prl8a2*) increased during pregnancy; an effect that was lost with PR knockout [70]. The increase in AIRE expression could therefore increase negative selection of T cells or augment Treg development to enhance maternal fetal tolerance, yet this remains to be studied. In vitro experiments and mouse models suggest that progesterone increases Tregs during pregnancy by progesterone binding to glucocorticoid receptor within thymocyte to decrease TCR signaling during negative selection [73, 74]. Loss of glucocorticoid receptors during pregnancy reduced Treg levels and increased pregnancy protection of EAE in mice [73].

##### 2.5.1.2 Peripheral Tolerance

Progesterone exerts broadly suppressive effects on the peripheral immune system with an inhibition of CD4^+^ Th1 responses and a shift toward Th2 immune responses [75]. This might help explain why certain autoimmune diseases, such as Myasthenia Gravis, often improve during pregnancy and relapse postpartum. In maternal PBMCs, progesterone treatment reduced the levels of proinflammatory cytokines IFNy, tumor necrosis factor (TNF)α, IL‐5, and IL‐10 in CD8^+^ T cells while increasing IL‐4. In the same study, the same T cells were less polyfunctional and decreased T cell proliferation [76]. Additional work comparing fetal cord blood to adult peripheral blood showed that progesterone promotes Treg differentiation and suppresses pro‐inflammatory Th17 development [77]. Consistent with this, pregnant mice exhibit progesterone‐driven expansion of Tregs, enhanced IL‐10 production, and increased suppressive activity [78]. Progesterone likewise attenuates autoimmune pathology in mouse models of autoimmune uveitis, where it diminished pathogenic Th17 cells and increased the function of Treg cells [79]. These progesterone‐mediated shifts in T cell balance support maternal‐fetal tolerance and dampen peripheral inflammatory responses [78]. More recently, Green et al. demonstrated that progesterone promotes Treg proliferation in the uterus of mice to support placental development and fetal growth [80]. In summary, progesterone has protective effects in pregnancy and autoimmune disease through expansion of Treg cells while suppressing pro‐inflammatory Th1 and Th17 effector subsets.

B cells express PR in addition to other sex hormone receptors [81]. While little is known about the direct effects of progesterone on B cells, as most experiments have been conducted during pregnancy when estrogen levels are also high, in vitro experiments of male and female leukocytes show a decrease in *BAFF* expression in males with progesterone administration [82]. Further, Hughes et al. showed attenuation of T cell‐dependent B cell antibody responses with global PR deletion, but not T cell‐independent antibody responses, suggesting indirect effects of progesterone on B cells via T helper cells [75]. In conclusion, progesterone is an important modulator of immune responses and more studies are needed to understand its many effects during and outside of pregnancy.

### 2.6 Menopause and Andropause

During natural aging, males and females experience a decline in gonadal sex hormones (Table 1). The loss of androgens in andropause in males is more gradual than the abrupt loss of estrogens associated with menopause in females. In addition to andropause and menopause, there are many iatrogenic scenarios where individuals might abruptly lose sex hormone production or have an alteration in hormone balance such as in ovarian cancer, testicular cancer, cancer treatments, certain medications, and premature ovarian insufficiency. Similarly, in females, menopause usually occurs between the ages of 45–55 years and is characterized by the sudden loss of estrogen and progesterone [35]. Menopause is defined as the first year after which no menses have occurred. Besides the critical loss of sex hormones, menopause and aging are associated with immunosenescence. Limited data suggest that the loss of estrogens may play a synergistic role with immunosenescence leading to a greater level of proinflammatory cytokines such as IL‐1, IL‐6, IL‐17A, and TNFa [35]. On the other hand, exogenous estrogens in females, either through oral contraceptives or hormone replacement therapy, are associated with increased inflammatory bowel disease (ulcerative colitis and Chron's disease) in women [83, 84, 85, 86].

Andropause is characterized by the gradual loss of testosterone in men with age and is associated with an increased risk of autoimmunity due to the loss of anti‐inflammatory effects of testosterone [34]. In contrast to menopause, andropause is associated with a more gradual decline in testosterone. There are limited studies on andropause, but as men age, there is an increase in Hashimoto's thyroiditis, SLE, MS, and RA, all female‐skewed autoimmune conditions [34, 47]. Similarly, men undergoing androgen deprivation therapy (ADT) for prostate cancer have an increased risk for RA, with longer exposure to ADT leading to increased risk for RA development [87]. Thus, hormonal shifts with natural aging might also be associated with equalization of autoimmune disease prevalence between males and females.

## 3 Sex Hormones and Immune Responses in Cancer

Many cancers have higher mortality rates in men, suggesting a potential sex‐linked difference in anti‐tumor responses [88, 89]. Immunotherapies, including immune checkpoint inhibitors (ICIs) such as anti‐PD1, have changed the face of cancer therapies and are now available to a large proportion of patients diagnosed with cancer. With the increasing use of immunotherapies as treatment for cancer, the effects of sex hormones on anti‐tumor immune responses, as well as potential autoimmune side effects of these treatments, are an important area of evolving research. This section will address recent developments in our understanding of the impact of sex hormones on anti‐cancer immunity, with a focus on androgens and estrogens.

### 3.1 Androgens as Suppressors of Anti‐Tumor Immunity

#### 3.1.1 Androgen Effects on T Cell‐Mediated Anti‐Tumor Immunity

ARs are present on multiple types of immune cells and contribute to immune modulation across tissues, as discussed above in the context of autoimmune diseases. As in autoimmune disease, androgens negatively regulate thymic T cell output and increase negative selection. In cancer, this may serve to reduce the T cell repertoire. In a recent paper by Polesso et al., androgen blockade increased thymus cellularity and release of recent thymic emigrants (RTEs) that migrated to tumors and produced a mature T‐cell like response in mice [90]. ADT triggered thymic epithelial cell (TEC) proliferation, increasing the production of RTEs that contributed to the anti‐tumor response. These findings suggest that androgen blockade effects on thymic output are an important mechanism by which anti‐cancer responses may be enhanced.

**FIGURE 2:**
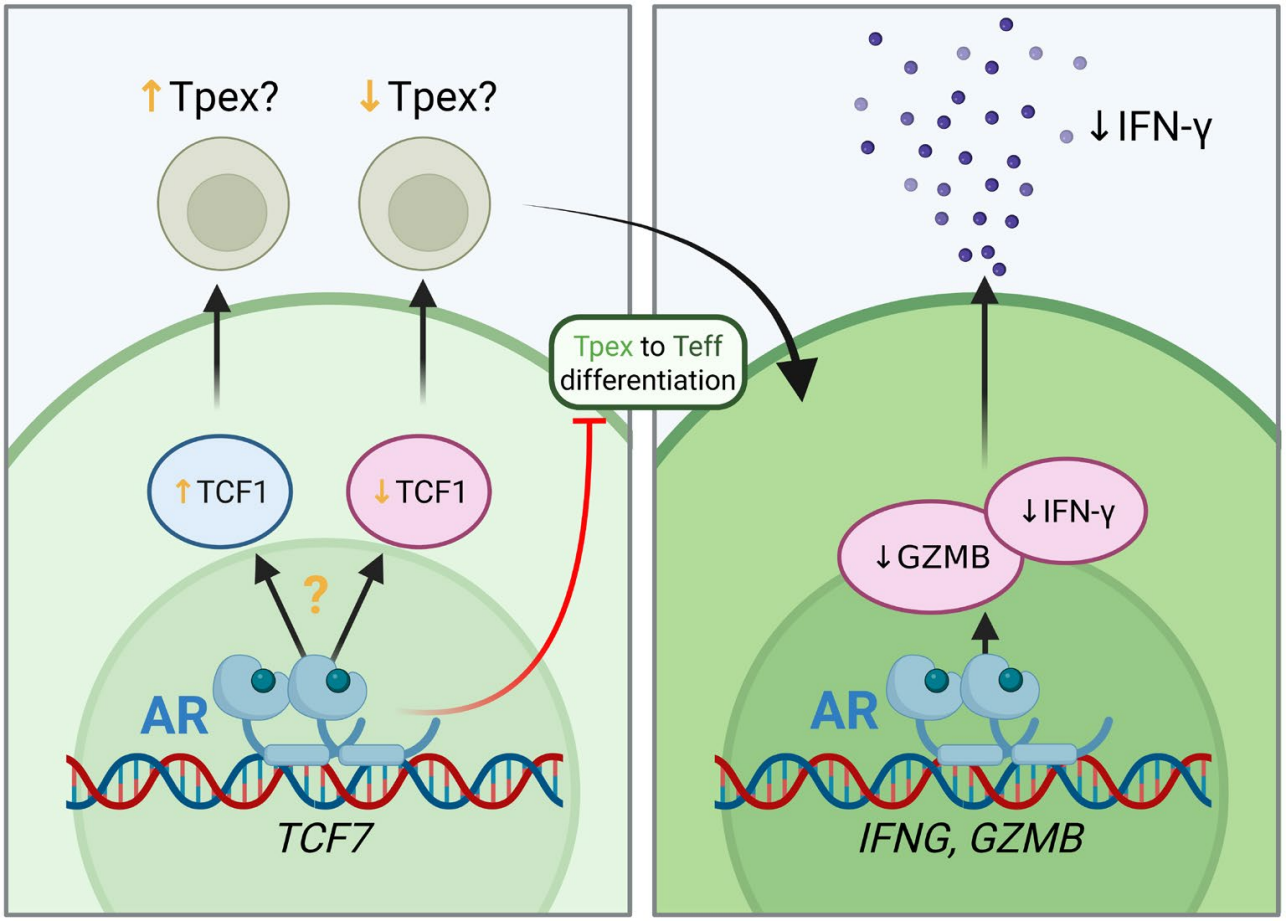
Direct effects of androgens on CD8^+^ T cell function. Androgens bound to androgen receptors (AR) have been reported to either increase or decrease *Tcf7* (which encodes TCF1) expression, with subsequent changes in frequency of stem‐like progenitor cells (Tpex). Conversion of Tpex to effector T cells (Teff) is also inhibited by androgen/AR complex. Androgen/AR complex can also decrease *Gzmb* and *Ifng* transcription in CD8^+^ T cells.

In addition to thymic mechanisms, androgens also shape T cell responses in the periphery. A recent report demonstrated that androgens exert their effects through direct inhibition of effector molecule expression. In the context of immune checkpoint inhibitor therapy, Guan et al. showed that androgen blockade increased cytotoxicity of CD8^+^ T cells and dampened tumor growth, suggesting a suppressive effect of androgens. They identified androgen response elements in the promoter regions of *Ifng* and *Gzmb*, and further showed that AR binding in the presence of androgens decreased *Ifng* transcription (Figure 2) [91]. Thus, androgens attenuate anti‐tumor immune responses through transcriptional modulation of effector molecules during checkpoint inhibitor therapy.

In addition, androgens may also attenuate anti‐tumor immune responses through their effects on *Tcf7*/TCF1^+^ progenitor exhausted CD8^+^ T cells (Tpex). This self‐renewing, stem‐like Tpex subset has recently been demonstrated to play a key role in anti‐tumor immunity. Tpex cells continually replenish the pool of cytotoxic effectors (Teff) as well as giving rise to exhausted cells (Texh) with limited effector capacity. Multiple groups have now shown that Tpex cells are the target of anti‐PD1 immunotherapy and that enhanced anti‐cancer immunity with anti‐PD1 immunotherapy requires *Tcf7*/TCF1 [92, 93].

Remarkably, sex differences in anti‐cancer immunity have been attributed to androgen effects on Tpex cells. Kwon et al. showed that the frequency of Tpex cells is increased in tumors of male mice and that this increase is androgen dependent. A sex‐specific regulon in Tpex cells was identified, and AR functioned as a direct transcriptional activator of *Tcf7* (which encodes TCF1). As a consequence, androgen serves to promote the progenitor phenotype and concurrently impair conversion of these cells to cytotoxic effectors with tumor killing ability [94]. Moreover, given the central role of Tpex cells in ICI therapy, androgens decreased effective anti‐tumor immune responses during ICI therapy in mice.

Yang et al. also reported that androgens altered the progenitor phenotype of intratumoral CD8^+^ T cells in male mice [95]. Similarly, androgens served as an impediment to effective ICI therapy in mice. However, in contrast to the Kwon et al. study, Yang et al. reported that the proportion of Tpex cells was decreased in tumors of male mice compared to females. This decrease in Tpex cells was accompanied by decreased tumor‐infiltrating IFNγ^+^ TNFα^+^ CD8^+^ Teff cells in male mice. Yang et al. also suggested that the observed differences in CD8^+^ T cell responses were due to androgen antagonism of TCF1^+^ expression. Thus, while multiple studies have shown that blockade of androgen signaling leads to favorable T cell effector differentiation and potentiates the efficacy of anti‐PD‐1 checkpoint blockade, the mechanism(s) by which this occurs remain controversial and will require further study (Figure 2).

#### 3.1.2 Androgen Effects on Innate Immune Cells in the Tumor Microenvironment

Innate immune cells are key components of the anti‐tumor immune response and are found within the tumor immune microenvironment. Specifically, tumor‐associated macrophages (TAMs) are closely connected with the development of the tumor microenvironment and can differentiate into heterogeneous subsets [96]. Androgens have been implicated in promoting an anti‐inflammatory phenotype in macrophages. This may occur through multiple mechanisms including the upregulation of IL‐10 [97]. In prostate cancer, Wang et al. reported that AR signaling acted as a transcriptional repressor for the pro‐inflammatory cytokine IL‐1β in TAMs [98]. Paradoxically, however, androgen blockade led to excessive secretion of IL‐1β and was associated with inhibition of CD8^+^ T cell function. Thus, while androgen blockade may have complex unexpected effects, androgens appear to enhance anti‐inflammatory cytokines (e.g., IL‐10) by TAMs while attenuating proinflammatory (e.g., IL‐1β) secretion. How these effects can be optimally targeted for cancer treatments requires further study.

NK cells also play a key role in cancer surveillance, and two recent studies suggest an indirect role for androgens in suppressing NK cell‐mediated anti‐cancer immunity [99]. Similar to T cells, NK cells express PD‐1 and are consequently regulated by changes in PD‐L1 expression on cancer cells [100]. Liu et al. found that androgens induce PD‐L1 expression in murine prostate and bladder cancers, limiting the efficacy of NK cell killing [101]. Blockade of androgen signaling resulted in decreased PD‐L1 expression on cancer cells, improving NK cell‐mediated anti‐cancer immunity. Similarly, Tang et al. noted that a high dose of androgens inhibited NK cell cytotoxicity to castration‐resistant prostate cancer [102]. This pathway increased PD‐L1 expression on tumor cells, triggering the PD‐1 inhibitory pathway in NK cells. This change was only observed in high doses of dihydrotestosterone (DHT), and low doses of DHT had little effect on NK cell killing. Of note, it has not yet been determined if there is a direct impact or influence of androgens on NK cell‐mediated anti‐cancer immunity.

### 3.2 Estrogen and Anti‐Tumor Immunity

Much research has been conducted on the expression of ERs on cancer cells, especially breast and ovarian cancer. In breast cancer cells, for example, the coexpression of ERɑ and ERβ by breast cancer cells was described as a positive indicator of tumor aggression [103]. As a consequence, estrogen receptor blockade (ERB) has long been a strategy to reduce tumor growth and recurrence in patients with ER^+^ breast cancer [104, 105]. On the other hand, how ERs affect anti‐cancer immunity is just beginning to be unraveled. The importance of understanding this is underscored by the precipitation of premature menopause and decreased levels of female sex hormones with the use of chemotherapy in women with breast cancer.

How changes in estrogen levels impact anti‐tumor immune responses remain poorly understood but recent studies have suggested improved anti‐tumor immunity with estrogen loss. In a mouse model of melanoma, Chakraborty et al. showed that estrogen signaling through ERα promoted macrophage polarization toward an immunosuppressive M2 phenotype. This effect on myeloid cells was correlated with increased CD8^+^ T cell exhaustion and resistance to checkpoint inhibitor therapy [106]. By contrast, treatment with the ER antagonist fulvestrant improved ICI tumor response in this model and was accompanied by an increased M1/M2 macrophage ratio and GZMB^+^ CD8^+^ T cells in the tumor [106]. Similarly, in other non‐ER^+^ murine tumor models (4T1 breast and CT26 colon), Kajihara et al. showed that ovariectomy or treatment with the ERα antagonist fulvestrant resulted in decreased tumor growth. Immune changes associated with estrogen blockade included decreased suppressive myeloid cell populations (i.e., M2 macrophages, myeloid‐derived suppressor cells) and increased effector T cells (i.e., IFNγ^+^ and GZMB^+^ CD8^+^ T cells) within tumors [107]. Consistent with these findings, Benslimane et al. reported improved tumor growth control and beneficial changes in the tumor immune microenvironment with estrogen loss/blockade in MC38 tumors metastatic to the liver and a murine pancreatic ductal adenocarcinoma tumor model [108]. Specifically, ovariectomy was associated with increased cytotoxicity of tumor‐infiltrating natural killer and CD8^+^ T cells and decreased accumulation of M2 macrophages in the tumor microenvironment. Finally, Benslimane et al. demonstrated an enhanced benefit of combining anti‐PD1 therapies with fulvestrant in these mouse models.

### 3.3 Sex Differences in IRAEs

Autoimmune side effects due to immune checkpoint inhibitor therapy (ICI), termed immune related adverse events (IRAEs), can occur in up to 70% of patients, depending on the treatment regimen used, and can affect multiple tissue subtypes. The most common organs affected are endocrine, gastrointestinal, skin, and pulmonary, but they can occur in almost every tissue. Compared to spontaneous autoimmunity, IRAEs occur rapidly within 4–8 weeks of the first ICI infusion and result in treatment discontinuation, hospitalization, and death. Some studies show increased risk of IRAEs in females, while others show no difference [109, 110]. Whether sex differences exist in IRAEs remains unclear and likely depends on organ type, cancer subtype, and patient hormone status. For example, in patients with NSCLC, premenopausal female patients were more likely to experience IRAEs compared to postmenopausal females and male patients, suggesting that higher estrogen levels may predispose to the development of IRAEs. In line with this, Zhang et al. demonstrate that, in checkpoint inhibitor induced myocarditis, induction of ERβ leads to downregulation of MANF and HSPA5 in female mice to increase ICI‐myocarditis in females [111].

Following the Keynote‐522 and ‐355 clinical trials, checkpoint inhibitor therapies are now first line treatments for patients with triple negative breast cancer [112, 113]. In addition, promising data in several recent trials (I‐SPY2, CheckMate 7FL, and KEYNOTE‐756) combining checkpoint inhibitor therapy with chemotherapy in patients with hormone receptor positive early‐stage breast cancer may portend even broader use of immune therapies in female‐specific cancers in the future [114]. Given the female predominance of breast cancer, this change in clinical practice will likely yield more data about sex‐linked differences in immunotherapy response and autoimmune toxicities.

## 4 Sex Chromosomes in Cancer and Autoimmunity

While differences in sex hormone levels underlie many sex differences in immunity, differences in sex chromosome complement also drive sexual dimorphism in the immune response. Most often, sex chromosomes consist of XX for females and XY for males, and this distinction has multiple downstream consequences. For instance, despite mechanisms in place to equalize dosage of X chromosomes between males and females, multiple sex chromosome‐linked genes are differentially expressed in female vs. male immune cells. Here we will review recent findings on how sex chromosomes contribute to sexual dimorphisms observed in anti‐cancer immunity and autoimmunity. These mechanisms include (1) the expression of Y chromosome genes in male cells; (2) loss of Y chromosome (LOY) that occurs in male cells; (3) expression of genes from the inactive X (Xi) chromosome in female cells, and (4) the expression of XIST by female cells. These mechanisms will be discussed in more detail below.

### 4.1 Expression of Y Chromosome Genes in Males

**FIGURE 3:**
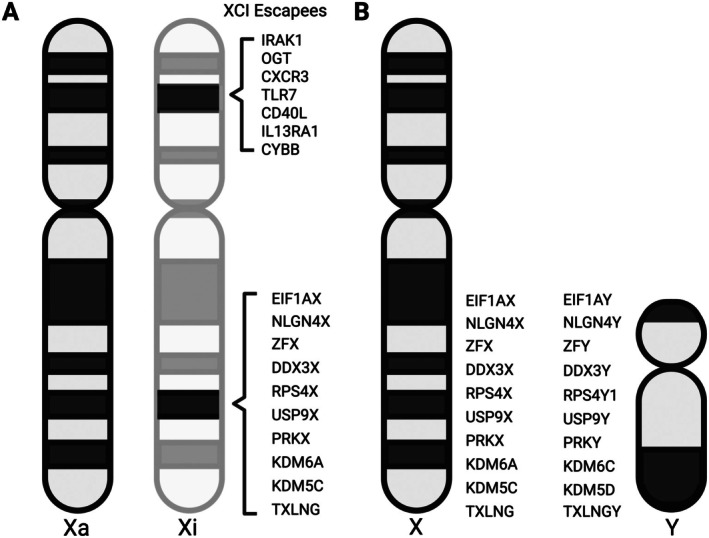
Genes expressed from both the Xi and Xa (XCI ‘escapees’) in female cells and homologous X and Y gene pairs. (A) Xi and Xa chromosomes with known immune‐related and Y homolog genes that escape XCI labeled. (B) Representative X and Y chromosomes with homologs labeled that are known to escape XCI in females.

The structure of the Y chromosome is quite complex and full of base pair repeats. As a consequence, the Y chromosome has been difficult to fully sequence and assemble, resulting in it being the last human chromosome to be completed. This recent achievement has contributed to our overall understanding of the Y chromosome and the genes it possesses [115]. While the Y chromosome is much smaller and contains fewer genes than the X chromosome, it contains 17 homologs to X chromosome genes (Figure 3B). These homologs (e.g., *KDM5D, KDM6C, DDX3Y*) encode for proteins with critical functions, and their X‐linked counterparts are often expressed from both the active X chromosome (Xa) and inactive X chromosome (Xi) in females (i.e., escape inactivation) [116].

The Y‐linked gene *Kdm5d*, for instance, encodes for an epigenetic regulator, and its X‐linked counterpart (*Kdm5c*) is expressed from both the Xa and Xi in females. Recently, *Kdm5d* was found to be a key driver of colorectal carcinoma (CRC) outcome and metastasis [117]. Deletion of *Kdm5d* improved tumor outcomes in mouse models, in part through indirect effects on the CD8^+^ T cell compartment. CD8^+^ T cells exposed to *Kdm5d* deficient cancer cells showed more efficient killing. Furthermore, constitutive expression of *Kdm5d* in cancer cells resulted in decreased CD8^+^ T cell infiltration. These findings suggest an immune mechanism by which expression of Y‐linked *Kdm5d* in CRC cells can contribute to increased metastases and mortality in males.

Male‐specific Y linked genes [e.g., sex determining region y (Sry)], which do not have X‐linked counterparts, may also underlie sex differences in anti‐cancer immunity. For instance, transcription factor Sry has been implicated in sex differences in cancer recurrence. Primary liver cancers occur more often in males after surgery, in both patients and mice [118]. Male mice had fewer CD8^+^ T cells and increased polymorphonuclear‐myeloid derived suppressor cells (PMN‐MDSCs) consistent with an unfavorable anti‐tumor immune response. Moreover, hepatocyte‐specific Sry overexpression in mice was found to be associated with postsurgical liver cancer recurrence whereas hepatocyte‐specific Sry deletion was protective. These findings implicate Sry in suppressing anti‐cancer immunity and unfavorable cancer outcomes. How Sry functions in hepatocytes to mediate these immune effects, however, will require further study.

### 4.2 Loss of Y Chromosome (LOY) in Males

**FIGURE 4:**
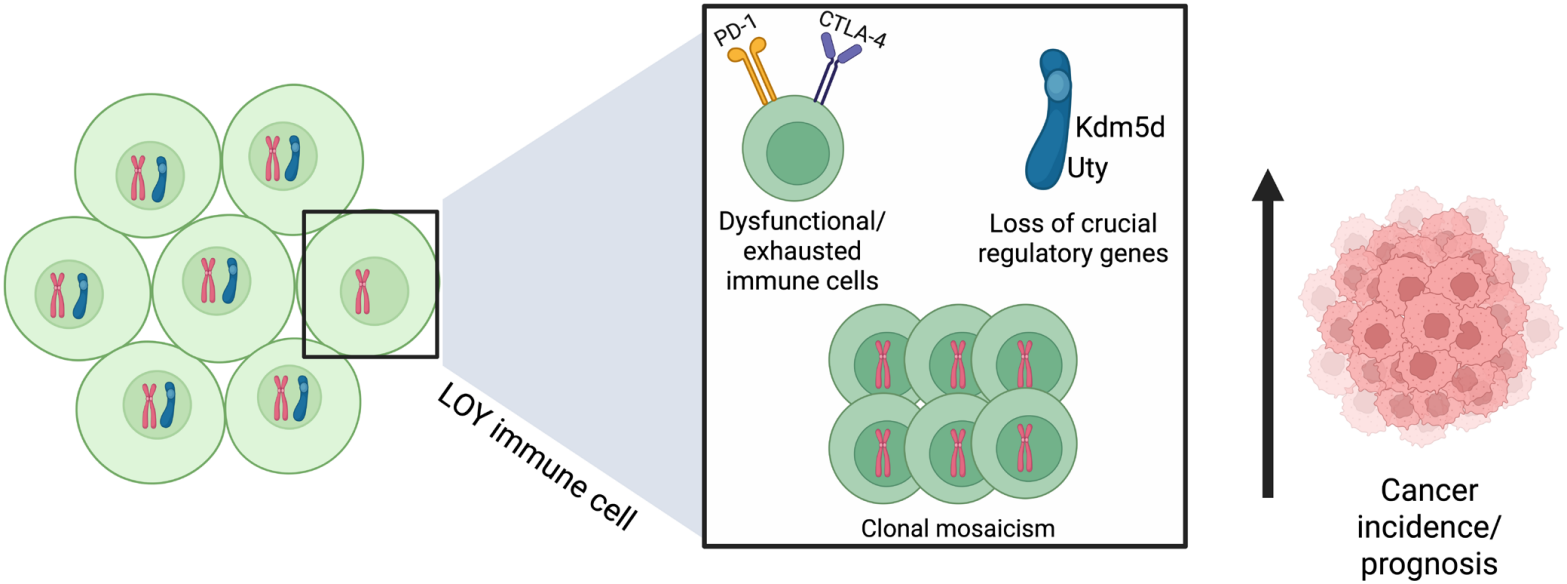
Consequences of LOY. LOY in immune cells can lead to dysfunction or exhaustion as seen in T cell subsets, overall partial or complete loss of important regulatory genes such as *Kdm5d* and *Uty*, and the further propagation of LOY. Together, these result in an increase in cancer incidence and poor prognosis for various malignancies.

Clonal mosaicism can occur in cells over time and accumulate in individuals with aging. The most common form of clonal mosaicism is loss of Y (LOY) chromosome in male cells (Figure 4), which is detectable in 20% of male peripheral leukocyte samples in the UK Biobank and 40% of samples in males over the age of 70 years [119]. Mosaic LOY (mLOY) in circulating leukocytes has been associated with multiple disease states including increased mortality, cardiovascular events, and other age‐related conditions [120]. While these associations are well established, a recent study has also demonstrated that mLOY is causal in promoting cardiac fibrosis with aging [121]. In a mLOY mouse model, LOY macrophages showed aberrant differentiation and increased pro‐fibrotic transforming growth factor (TGF)‐β production in the myocardium. Thus, LOY immune cells have a pathogenic capacity in age‐associated disease.

Emerging evidence also suggests that LOY may confer pathogenic capacity to immune cells beyond myeloid cells. A recent analysis of scRNA‐seq datasets from liver, lung, and colorectal cancer patients revealed that regulatory T cells (Tregs) have a marked increase of LOY and comprise a higher proportion of CD4^+^ T cells compared to other T cell subtypes [122]. LOY Tregs exhibit alterations in the expression of multiple genes, with many genes having known immune functions. How LOY may affect Treg suppressive function and the mechanisms by which these changes can alter cancer susceptibility remain to be determined.

While these findings suggest that LOY in immune cells may have effects on their function and phenotype, recent work has also highlighted an indirect mechanism by which LOY in cancer cells impacts immune cell function [123]. In bladder cancer, LOY in cancer cells was associated with increased tumor burden in immune‐competent mice. Remarkably, this increased tumor burden was not due to increased growth by LOY cancer cells, since LOY cancer lines did not proliferate more than control cancer lines. Moreover, faster growth was also not seen when LOY cancer cells were implanted into immunodeficient *Rag2* ^−/−^ *Il2rg* ^−/−^ mice, suggesting that increased tumor burden was due to increased efficiency of LOY tumors in evading anti‐tumor immunity [123]. Y chromosome genes *Kdm5d* and *Uty* were found to be crucial to tumor growth, as single knockouts of each in cancer cells significantly increased tumor growth while overexpression diminished tumor size. LOY tumor cells displayed more dysfunctional and exhausted CD8^+^ T cells (i.e., higher levels of TOX expression), suggesting that LOY in cancer cells alters T cell function in the surrounding tumor microenvironment.

More recently, LOY in cancer cells has been proposed to be linked to LOY in neighboring T cells, and this concurrent LOY impacts cancer outcomes [124]. A comprehensive analysis of bulk‐ and single‐cell RNA sequencing datasets from 29 different human cancers revealed LOY not only in malignant cancer cells but also in tumor stromal and immune cells [124]. LOY had the greatest impact on CD8^+^ and CD4^+^ T cell transcriptomes, increasing immunosuppressive and exhaustion signatures while decreasing cytotoxicity signatures. While more studies are needed to fully understand the mechanisms by which LOY spreading might occur, concurrent LOY in benign and malignant cells was associated with poor survival in cancer patients.

In addition to anti‐cancer immune responses, LOY in circulating immune cells has also been implicated in predisposition to autoimmunity. Previous studies have noted associations between autoimmune thyroiditis in male patients and LOY in peripheral blood [125]. However, the mechanism by which LOY may enhance autoimmunity is not currently clear. Interestingly, polymorphisms in Y chromosome genes have been reported to alter autoimmunity predisposition in multiple mouse models of autoimmunity and to modify sexual dimorphism seen in these models, suggesting possible mechanisms by which LOY may enhance autoimmune capacity [126].

### 4.3 Expression of Genes From the Inactive X (Xi) in Female Cells

Females undergo a process known as X chromosome inactivation (XCI) to achieve X‐linked gene dosage compensation. This process is driven by the long non‐coding RNA (lncRNA) Xist, which initiates the process of XCI by coating the randomly selected future Xi and propagating epigenetic and transcriptional changes that result in a silenced X chromosome [127]. Importantly, however, 20%–30% of X chromosome genes in humans are still expressed at varying degrees from the Xi [128]. As a consequence, genes that are expressed from both the Xa and Xi in females (also known as XCI “escapees”) have sexually dimorphic expression, with higher expression in XX females compared to XY males (Figure 3A). Although to a lesser extent, biallelic expression of X‐linked genes also occurs in mouse cells, with about 3%–7% of genes exhibiting expression from the Xi [129]. Thus, biallelic expression of X‐linked genes in females is a conserved mechanism underlying sexual dimorphism across species.

Among the XCI “escapees” in humans and mice are epigenetic regulators that play critical roles in immune cell function and may underlie sexually dimorphic responses in autoimmunity. For instance, the histone 3 lysine 27 (H3K27) demethylase *Kdm6a* (which encodes UTX) has been implicated as an XCI escapee whose higher expression level in female CD4^+^ T cells and microglia is responsible for female bias in the incidence of MS [130, 131]. Interestingly, deletion of UTX in microglia protected female mice in a mouse model of but did not have an impact on male mice.

In addition to MS, UTX has also been implicated in regulating immune cell function in other female‐biased conditions. In SLE, for example, pharmacological inhibition of UTX (with GSKJ4) in SLE patient monocytes resulted in decreased interferon‐stimulated gene (ISG) expression [132]. Similarly, GSKJ4 treatment in a resiquimod (R848) mouse model of SLE resulted in decreased production of autoantibodies as well as lower ISG expression. While these findings suggest a potential role for UTX, it is important to note that GSKJ4 is not a specific inhibitor of UTX but instead is an inhibitor of the histone demethylase activity of both UTX and JMJD3 (encoded by *Kdm6b*). Thus, more specific approaches for inhibiting UTX are needed to attribute these findings to UTX. Additionally, in a mouse model of colitis, T cell specific deletion of UTX protected mice from the development of autoimmune colitis [133]. Together, these recent findings support the idea that higher levels of UTX in females can lead to female‐biased incidence of autoimmune diseases.

At the same time, a recent report suggests an opposing role for the X‐linked histone 3 lysine 4 (H3K4) demethylase *Kdm5c* (which encodes Jarid1c) [134]. Similar to UTX, Jarid1c is expressed from both the Xa and Xi in humans and mice. Doss et al. utilized a male‐biased adoptive transfer model of EAE to show that overexpression of Jarid1c in Th17 cells protected against disease transfer [134]. These findings suggest that Jarid1c negatively regulates pathogenic Th17 cells in this model and is therefore protective in Th17‐mediated autoimmune conditions. Indeed, although the incidence of MS is higher in females, males with MS have a more severe disease course. Thus, these findings implicate lower Jarid1c expression in male Th17 cells as a mechanism underlying increased MS severity in males.

In addition to autoimmunity, accumulating evidence suggests a key role for XCI escapees in anti‐cancer immunity. Dysregulation of XCI, inferred using an X‐Reactivation (X‐Ra), in circulating monocytes, peripheral blood, and other cell types has been correlated with triple‐negative breast cancer and worse survival [135]. Additionally, specific XCI escapees have been implicated in the control of anti‐cancer immunity. For example, UTX has been implicated in the sex‐biased immune response against glioblastoma [136]. Female mice had higher survival in a mouse model of glioblastoma due to a more robust T cell response. Specifically, females had fewer progenitor exhausted T cells (CD8^+^ CD44^+^ PD1^+^ TCF1^+^ TIM3^−^) and increased effector T cells (CD8^+^ CD44^+^ TCF1^−^ TIM3^−^). Treatment of mouse CD8^+^ T cells with the pharmacological inhibitor GSKJ4 led to decreased expression of IFNγ and increased expression of the exhaustion marker TIM3, suggesting that higher UTX expression may underlie the more robust anti‐cancer T cell response in females. As noted above, however, GSKJ4 inhibits the histone demethylase activity of both UTX and JMJD3, so a more specific approach to UTX inhibition is needed to define UTX's role in the female‐biased immune response against glioblastoma.

In support of a role for UTX in promoting anti‐cancer immunity, T cell‐specific UTX deletion has been reported to decrease the immune response against colon cancer [137]. Additionally, multiple studies have now reported that UTX mutations in cancer cells can alter tumor‐infiltrating immune cell populations. In bladder cancer, for instance, low UTX expression due to UTX mutations in cancer cells was correlated with decreased immune cell infiltration in the tumor [138]. Moreover, UTX‐deficient bladder cancer cells have also been reported to increase production of cytokines important in the polarization of macrophages to the M2 lineage [139]. Thus, UTX may alter anti‐cancer immunity both through its direct effects in T cells and through its indirect effects in cancer cells.

### 4.4 XIST Expression in Female Cells

As discussed above, the long noncoding RNA (lncRNA) Xist directs XCI in female cells. Consistent with this role, perturbation of Xist in B cells released X‐linked genes (i.e., *TLR7*) from inhibition. As a consequence, Xist deletion in B cells was associated with increased formation of pathogenic CD11c^+^ atypical B cells and a higher incidence of SLE and RA [140]. In addition, mild global Xist deficiency was induced in mice due to a mutation in *Ftx*, an X inactivation center gene. These Xist‐deficient mice also reactivated gene (i.e., TLR7) expression from the Xi in immune cells, with subsequent dysregulation in B cells, dendritic cells, and monocytes [141]. Mild Xist‐deficiency in mice led to the spontaneous development of inflammatory signals and symptoms consistent with SLE. These studies highlight the importance of Xist‐mediated TLR7 repression in maintaining tolerance and preventing systemic autoimmune conditions such as SLE.

In T cells, Xist has been shown to dynamically regulate XCI with stimulation [142]. In unstimulated T cells, the Xi was found to be transcriptionally silent with an enrichment of repressive histone marks. After stimulation, T cells acquired epigenetic modifications on the Xi that poised certain genes for expression. Xist localization was found to be dependent on NF‐κB signaling, as both treatment with NF‐κB inhibitors and a genetic murine T cell‐specific knockout of NF‐κB kinases resulted in decreased Xist cloud formation. Interestingly, this appears to be lymphocyte specific, as similar reductions in Xist cloud formation were not observed in a murine embryonic fibroblast line. Taken together, these findings demonstrate the importance of Xist in repressing X‐linked genes in immune cells and define the molecular underpinning of Xist‐mediated repression of X‐linked genes.

Notably, Xist has recently been shown to have roles beyond XCI. For instance, Xist has been shown to dampen gene expression not only on the X chromosome but also on autosomes [143]. Xist RNA antisense purification (RAP‐seq) was used to identify autosomal targets of Xist, and the expression of these autosomal gene targets was lower in females than males. Because these findings were made in human pluripotent stem cells (hPSCs) and murine PSCs, it remains unclear whether Xist also regulates autosomal gene expression in immune cells. If shown empirically to occur, this non‐canonical Xist function may further explain sex differences in immunity through alterations in autosomal gene expression.

**FIGURE 5:**
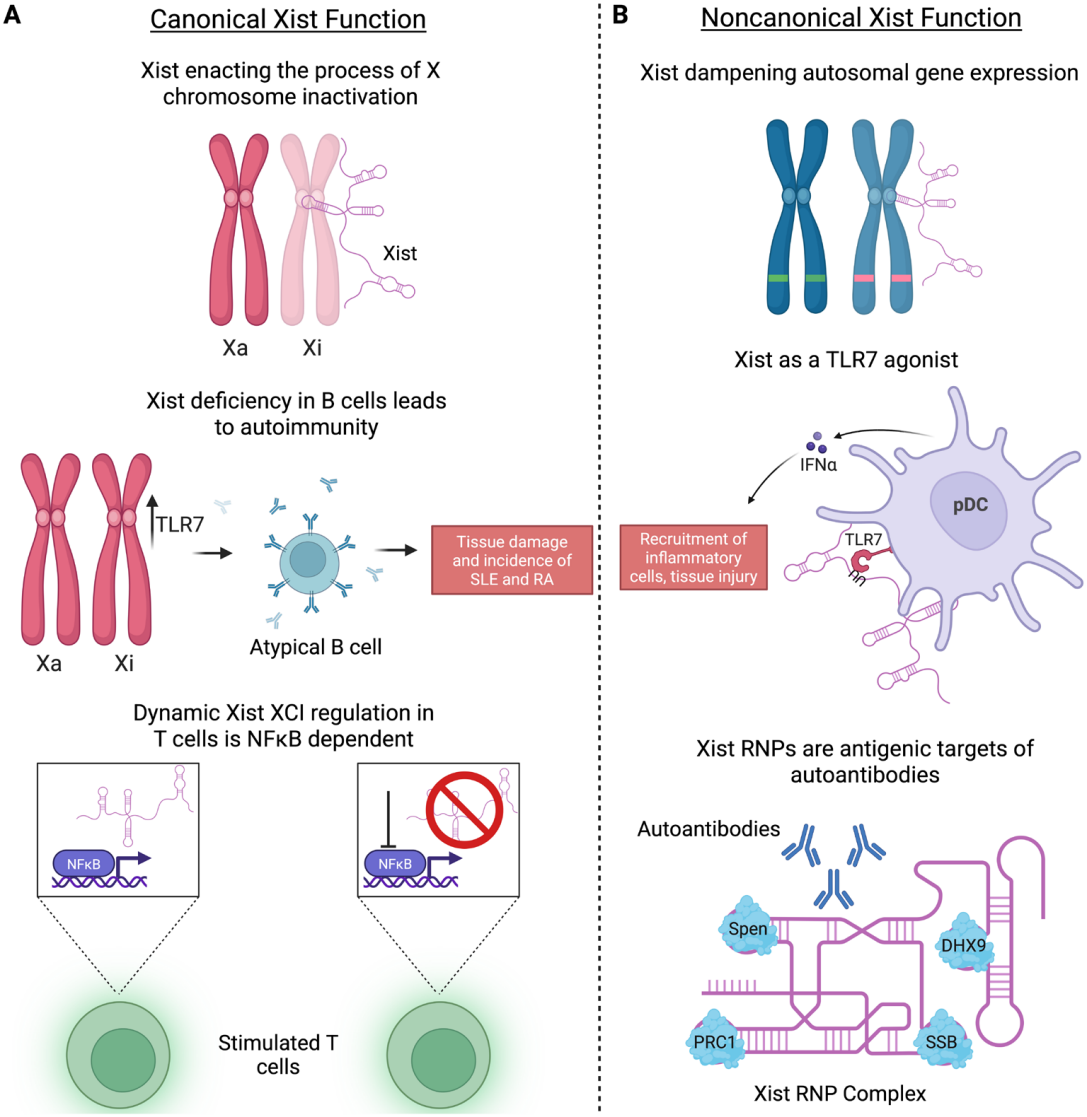
Canonical and noncanonical functions of Xist. (A) Canonical Xist function of coating the Xi and enacting the process of XCI. Absence of Xist has been shown to lead to increased autoimmunity through increased TLR7 expression from the Xi. (B) Noncanonical functions of Xist. Xist has been shown to dampen autosomal gene expression and function as a TLR7 agonist to promote incidence of autoimmunity. Xist can also form RNP complexes (with proteins such as Spen, PRC1, DHX9, and SSB) that are antigenic and are targeted by autoantibodies in systemic autoimmunity.

In addition to autosomal gene regulation, other “non‐canonical” Xist functions have now been delineated which may contribute to immune activation (Figure 5). First, Xist RNA was found to be a TLR7 ligand that can stimulate pDCs in patients with SLE [144]. Xist contains a motif (UU‐containing sequence) that TLR7 preferentially binds to, leading to its induction. Transfection of pDCs with the sequence of Xist containing the TLR7 agonist motif led to robust IFNα production. Depletion of Xist significantly reduced the amount of TLR7 ligands present in cells. Furthermore, Xist expression was shown to be higher in SLE patient PBMCs compared to healthy controls.

Second, Xist complexes with multiple ribonucleoproteins (RNPs), and these RNPs serve as autoantibody targets to promote autoimmunity in a SLE mouse model [145]. In SJL/J male mice, inducible expression of Xist was associated with higher autoantibody production and more severe lupus‐like disease. Furthermore, the T and B cell compartments of male mice expressing Xist were reprogrammed to have decreased immune modulatory programs and increased gene signatures associated with female mice.

## 5 Conclusions

Much progress has been made in recent years in understanding the biological underpinnings of sex differences in immunity. A major development has been an appreciation for sexually dimorphic changes that occur across the lifespan. Understanding the effects on immune responses of hormonal changes that occur over time, for instance, is an important step toward optimizing immune health in individuals undergoing menopause or andropause. Additionally, a recognition that somatic mosaicism accumulates with age and plays a role in altering the immune response is also an important step toward improving the immune response in cancer and tolerance toward self‐antigens with aging. Finally, with the expanding number of immunotherapies being utilized in the treatment of cancer and autoimmunity, how chromosomal complement and hormonal levels interact with these immunotherapies is a subject of much interest. A deeper understanding of these interactions will allow the maximization of clinical benefit while minimizing adverse outcomes in individuals eligible for these therapies.

## Funding

This work was supported by the National Institute of Allergy and Infectious Diseases (Grants 5R01AI174519‐03, 5R01AI174519‐03S1, 5U19AI181729‐02).

## Conflicts of Interest

The authors declare no conflicts of interest.

## Data Availability

Data sharing not applicable to this article as no datasets were generated or analyzed during the current study.
